# A Comparative View of the Modern Medical Theories, and Their Agreement with Practical Facts and Observations

**Published:** 1799-06

**Authors:** N. P. Gilbert


### *A Comparative View of the Modern Medical Theories, and their Agreement with Practical Facts and Obfervations.*— *By* N. P. GILBERT.

[A Memoir read at the Meeting of the Medical Society of Paris, Dec. 12, 1798.]

IF it be true that health is the firft of bleffings, the art of preferving it, and reftoring it when impaired by difeafe, is undoubtedly the moft ufeful of arts. This may be defined the fcience of obfervation, and that of availing ourfelves of favourable opportunities in regulating the functions of the human body. Since the progrefs of the *curative art* is directed by this double guide; fince the knowledge of paft ages is not loft to the prefent; its laws are clear, its precepts eafy to be comprehended, and their application often fuccefsful. If we confult the annals of the infancy of fcience, we fhall find, that the firft phyfician was inftinct, and the firft elements of medicine were the leffons of nature, gradually ftrengthened by thofe of experience. It is impoffible to reflect, without being fenfibly ftruck, on the early hiftory of medicine. The fick were expofed in the temples, or other public places; the citizens began the work of the day, by hofpitable vifits to thefe retreats of diftrefs. Perfons who had been afflicted with maladies fimilar to thofe of the objects of their vifit, adminiftered the remedies which they had found of advantage in their own cafes. Thus did men profit by the experience of each other. This general and lively fympathy, thefe affecting fraternal attentions, this religious anxiety to relieve our fellow-creatures, had an inexpreffible attraction, which cannot but excite our regret for the difufe of this ancient cuftom. Happy golden age! in which the delightful union of phyfical frugality, and moral purity of life, extended a healthy and tranquil ftate of man to the period affigned by nature; and left to that provident and wife mother all the energy which fhe required to counteract teract with succefs the deftructive influence of external agents! Civilization daily increafed the wants and the paffions of men; they foon abandoned experimental medicine, which, indeed, was obliged to creep along from experiment to experiment, often unfuccefsfully; but for thefe they fubfti-tuted a hypothetical fyftem of medicine, which did not fail continually to miflead them. HIPPOCRATES arofe, who, if he was not the creator of medical fcience, may unqueftionably be confidered as its father and legiflator. This great man immediately wrefted medicine from the hands of the priefts, who had made it a matter of religion, enveloping it in the thick veil of myftery and fuperftition. He collected the principal difcoveries handed down by tradition; he fubmitted facts to the teft of found reafoning, and founded the fcience upon the ruins of thofe fanciful fyftems that had pre-ceded it. Whence is it, that fo bright an example has found fo few imitators? By what fatality was it, that the path of practical medicine, fuddenly raifed by the genius of this celebrated legiflator to an eminent degree of perfection, was fo foon abandoned, and would have fallen into difgraceful oblivion, if a few men of enlightened minds had not, from age to age, been at pains to preferve it?

Thefe deplorable errors have, in all ages, been the produce of fanguine and ambitious men. Led aftray by too lively an imagination, ftrongly influenced by the philofophical opinions of the day, they fubftituted the glitter and luxury of fcience, for fimple facts and obfervations; they created oppofite fyftems; founded inimical fects; furrendered medicine to theories generally unfounded, and enveloped experience in impenetrable clouds.

However, in eftablifhing the principle, that all fyftems have retarded the progrefs of the healing art, let us do juftice to the intentions of truly eftimable phyficians, who have been driven into that vortex: what other objects could they have, than to perfect the fcience, facilitate its ftudy, and fimplify its practice?

Of this truth we fhall be convinced, if we obferve, that every fyftem is not fo much the error of the innovator, as of the age in which he lived. Moft medical theories owe their origin to the rapid movement which is fo general and irrefiftible among men of talent, in favour of a predominant opinion.

It muft be granted, that there has not probably been one fyftem of medi-cine which, however dangerous from its indifcriminate application, has not been productive of interefting practical obfervations, valuable facts, and even frequently beneficial medical precepts.

It would therefore be an ufeful work, to enumerate and defcribe all thefe different theories, fo as to advance the progrefs of practical medicine. Such a work * would be extremely interefting, particularly at this time, when a new fyftem of medicine, after having enjoyed high reputation in England, Germany, and Italy, is feeking to naturalize itfelf in the foil of this immenfe republic; at this time, when the brilliant difcoveries of modern chemiftry tend, perhaps, to produce in the fchool of medicine a revolution more complete than any that has heretofore taken place. Will the healing art gain or lofe by the eftablifhment of thefe new medical theories?—how ought they to be received by phyficians, anxious to perfectionate the art?—how far ought they to have a place in the prefent plan of ftudy?—what part ought to be taken in the midft of their enthufiaftic fectaries, and their obftinate antagonifts?—Such are the queftions which the medical philofopher will propofe to himfelf in his ftudy. The folution of thefe problems demands, no doubt, the moft profound deliberation. I feel how much fuch an undertaking is beyond my abilities. I fhall, however, attempt to trace the outlines of the inquiry, leaving it to a more experienced pen to finifh the picture.

The doctrine of Stoll; the theory of Brown; the application of the principles of modern chemiftry to the art of healing, or chemical pathology; —thefe are the theories which I purpofe fuccinctly to analyfe. I fhall compare them with each other, and confider their greater or lefs influence on the practice of medicine.

The principles of the humoral pathology, eftablifhed by obfervation, and confecrated by a judicious and fuccefsful practice, the powerful influence of the *annual conftitutions*, and of the periodical return of the feafons, upon the generation and fucceffion of febrile difeafe;—fuch are the foundations of the doctrine of Stoll. It is not a theory or a fyftem: the fyftems of medicine have uniformly been founded upon an hypothefis or abftract principle; the doctrine of Stoll refts upon facts. Confult his clinical annals (*Ratio medendi*): who deferves better of medical fcience, if allowance be made for the difgufting prolixity which characterifes the greater part of fcientific and literary German works? Who furnifhes the young practitioner with better advice, to confine himfelf to a general rational method, when the curative indications are not obvious and fatisfactory? Who deprecates more ftrongly fubmiffion to hypothefis and opinion?

Stoll was the man who eftablifhed the principle, or rather, who revived the doctrine of Hippocrates, tending to prove that the feafon of the year determines the production of the material caufe of fevers, while their form and feat depend upon the pre-difpofing caufes, and the mode of life adopted by

* It already exists in "Sprengel's *History of Medicine*," 3 vols. 8vo. (in German.) See No. I. p. 83, of this Journal.

by the individual; hence several diseases which appear very distinct from each other by their symptoms, are analogous in their causes, and yield to a similar treatment.

Thus far have we been able to recognise in the clinical practice of Stoll, a physician truly Hippocratical, and guided by observation. Why has he not followed the same method in the precepts he has given us? Why, after having declared himself the enemy of theories, has he, in his aphorisms, adopted the theory of Boerhaave? It is indeed modified, reformed, and in many points, not essentially different from the present state of the science; yet the laws of physics and mechanics are still there applied, without qualification, to explain the phenomena of health and disease; humoral pathology is throughout predominant; he takes no notice of that sublime doctrine of the *vital principle* and *organic movements*, which are so well unfolded in the writings of BORDEU. The 'Aphorisms' of Stoll, perhaps the only elementary medical book now extant, ought then to be read with due precaution. If put into the hands of the young practitioner, ever eager in the pursuit of science and erudition, ever anxious to explain facts, it may divert him from the practical study of medicine, prejudice his mind in favour of a system, and conduct him to fatal deviations; unless the prudence of a judicious interpreter, the talents of an enlightened and philosophical commentator, distinguish in this work between that which belongs to the medical dogmas, to the doctrine of Hippocrates, and that which is merely the result of an ingenious theory.

Before entering upon the examination of the new modern systems, it may be proper to take a general view of the state of medicine in Europe, about the middle, or towards the end, of the present century.

(To be resumed in our next.)

